# Curcumin-loaded nanocarriers for dermatological applications: current advances and biomedical implications

**DOI:** 10.17179/excli2026-9317

**Published:** 2026-05-25

**Authors:** Tanuj Sharma, Jagdeep Singh, Vishal Sharma, Diksha Sharma, Rina Das, Dinesh Kumar Mehta, Isha Chawla, Sonia Yadav, Shivani Chopra, Hitesh Chopra, Jai Bharti Sharma

**Affiliations:** 1 M.M. College of Pharmacy, Maharishi Markandeshwar (Deemed to be) University, Mullana, Haryana, India; 2 Department of Pharmaceutical Chemistry, Swami Devi Dyal College of Pharmacy, Barwala, 134118, India; 3 School of Medical and Allied Sciences, K.R. Mangalam University, Gurugram 122103, Haryana, India; 4 Department of Biosciences, Saveetha School of Engineering, Saveetha Institute of Medical and Technical Sciences, Chennai - 602105, Tamil Nadu, India; 5 Centre for Research Impact & Outcome, Chitkara College of Pharmacy, Chitkara University, Rajpura, 140401, Punjab, India

**Keywords:** topical drug delivery, nanoparticles, anti-inflammatory, antioxidant, antibacterial, nanocurcumin

## Abstract

Topical drug delivery routes have long served as a primary therapeutic approach for treating various skin disorders. However, conventional formulations, like creams, ointments, and gels, have a number of drawbacks, such as systemic toxicity, short duration of action, low skin permeability, and drug instability. Through the advancement of innovative delivery carriers such solid lipid nanoparticles, nanostructured lipid carrier system, nanoemulsions, & transdermal patches, recent developments in nanotechnology have completely changed topical delivery. These technologies improve skin penetration, provide regulated release, and increase medication absorption. Furthermore, because of their excellent therapeutic results, tailored particle size, and high entrapment effectiveness, formulations such as cubosomes, nanogels, and microsponges are being investigated. The significant expansion of topical delivery platforms, particularly hydrogel-based and nanoemulgel formulations, is highlighted by biostatistics from 2023-2024. Novel delivery systems have shown considerable effectiveness in treating dermatological disorders such atopic dermatitis, psoriasis, and eczema in clinical trials. Concurrently, the anti-inflammatory, antioxidant, and antibacterial qualities of botanical substances such as curcumin, bolstered by improved delivery through liposomal and nanocurcumin formulations, have demonstrated encouraging outcomes. This review explored the advancement of nanotechnology-enabled topical and transdermal delivery platforms, their clinical use, technical developments, and the growing use of natural bioactives like curcumin in dermatological treatment are all thoroughly covered in this study. The combined results highlight how crucial novel formulations and delivery methods are to resolving issues with the skin barrier and enhancing patient outcomes.

See also the graphical abstract(Fig. 1).

## Introduction

The skin, recognized as the body's largest organ provides multilayered protection through chemical, immunological, microbiological, and physical barriers, all of which are essential for preserving homeostasis (Norlén, 2023[91]). These protective layers control internal physiological equilibrium in addition to shielding the body against outside threats. The stratum corneum, the outermost layer of the skin, functions as the primary barrier regulating topical and transdermal drug permeation in therapeutic management. The drug's capacity to pass through this barrier and get to the targeted site of action is significantly influenced by its physical & chemical characteristics, i.e solubility, lipophilicity, molecular size, & ionic charge (Valecha et al., 2023[140]). Topical drug delivery system is a localized treatment approach, wherein drug formulations are directly applied to the skin for managing cutaneous conditions. According to de Souza et al. (2020[40]) and Mohd Nordin et al. (2021[90]), although a variety of conventional topical medicines are commercially available; these formulations frequently have drawbacks such drug instability, limited duration of action, low penetration, and skin irritation. Transdermal systems are further limited by the skin's effective barrier function, which permits only tiny, lipophilic molecules to get through. In order to tackle the associated challenges, recent developments in pharmaceutical nanotechnology have produced delivery systems based on nanocarriers, including nanostructured lipid carriers (NLCs), solid lipid nanoparticles (SLNs), nanoemulsions, & nanovesicles. These systems are intended to improve the therapeutic index, skin retention, & systemic availability of poorly water-soluble drugs. Transdermal patches, microsponge delivery devices, and nanogels sub-100 nm particles that provide targeted and regulated drug release are further advancements (Sharma et al., 2024[123]; Mendake et al., 2024[84]). 

The market for topical drug delivery has grown quickly due to advancements in formulation science and the rising demand for non-invasive treatments. For example, the hydrogel-based drug delivery market was estimated at USD 6,415 million in 2021 and is projected to grow at a compound annual growth rate (CAGR) of 7.6 %, reaching USD 12,357 million by 2030 (Shukur et al., 2025[125]). With over half of the top 20 best-selling pharmaceuticals medications being biopharmaceuticals, the biopharmaceutical industry which is closely related to topical delivery systems was expected to reach $388 billion in 2024 (Kirkby et al., 2020[67]). This momentum is also evident in research outputs, since drug delivery is linked to 59 % of related patents and 76 % of publications in nanomedicine (Bosetti, 2015[29]). Since superficial fungal infections are the fourth most prevalent type of illness, affecting over 40 million individuals worldwide, topical formulations are very important in treating them (Bolla et al., 2019[28]). Superior results have been obtained with advanced nanoformulations; after 24 hours, nanoemulgels containing eucalyptus and clove oils had drug releases of 89±7 % and 91±4.5 %, respectively. Vesicle diameters of 150.6±11.1 nm were reported by niosomal gels, together with 92.41±2.44 % release of drug & 92.36±2.85 % encapsulation efficiency. Similarly, cubosomes showed 61.66±1.01 % entrapment efficiency with much higher AUC values than commercial products, while microsponge-based gels showed 84.19 % drug release over 24 hours (Ramesh et al., 2024[111]; Shoman et al., 2023[124]). These statistical achievements reflect the industry's commitment to developing more effective topical delivery systems with enhanced patient compliance and therapeutic outcomes. Conventional cutaneous delivery systems encompass a wide variety of topically administered therapeutic formulations intended to exert local or systemic pharmacological effects. The most utilized semisolid preparations include creams, gels, and ointments, which serve as the primary vehicles for topical drug administration (Weiss, 2011[144]). Creams are O/W or W/O emulsions that govern good spreadability & patient acceptance, while ointments are typically lipophilic bases offering prolonged contact time and occlusive properties (Barnes et al., 2021[23]). Gels, which are cross-linked polymer networks swelled with a liquid, have gained widespread recognition due to their smooth, non-greasy texture, easy spreadability, and superior application properties compared to traditional creams and ointments. Additionally, liquid preparations such as lotions, solutions, and suspensions are widely employed for their ease of application over large surface areas (Bhuyan et al., 2021[26]). Beyond traditional dosage forms, transdermal patches, foams, sprays, aerosols, nail lacquers, film-forming systems, and emulgels are tailored for site-specific applications and enhanced patient compliance (Prausnitz and Langer, 2008[108]; Tapfumaneyi et al., 2022[133]). In 2023 and 2024, there has been a lot of action in the clinical trial landscape. The majority of the 486 clinical trials on nanoparticle-based delivery that were documented between 2002 and 2021 involved liposomes (44 %) and protein-based systems (26 %), according to Namiot et al. (2023[88]). Between September 2023 and January 2024, 25 active trials (11 Phase II, 14 Phase III) for atopic dermatitis were found (Patel et al., 2024[95]). Dissolution microneedles exhibited ground-breaking efficacy for long-term dermatological therapy, whereas novel monoclonal antibody therapeutics indicated significant decreases in EASI scores (Priya et al., 2023[109]).

One important molecule among herbal actives that has demonstrated therapeutic advantages for psoriasis, eczema, acne, hyperpigmentation, skin cancer, and wound healing is curcumin, which is found in turmeric. Anti-inflammatory, antioxidant, antimicrobial, and signaling regulation processes involving the MAPK, PI3K/Akt, and JAK/STAT pathways promote its broad-spectrum action (Koch et al., 2023[69]). Hydrogels, creams, liposomes, and nanocurcumin have all been used to improve its distribution. Clinical adherence is still difficult even with promising treatments. Only 30.9 % of patients with actinic keratosis adhered to the suggested topical treatment, indicating 46.9 % non-adherence (Koch et al., 2023[69]). However, because of their increased efficacy and user compliance, emulgel-based formulations have demonstrated clinical and commercial potential (Kumbhar et al., 2025[70]). For antifungal medications such amorolfine and terbinafine, laser-assisted drug delivery techniques employing CO_2_ and Er:YAG lasers showed greater drug penetration and results (Tartaglia et al., 2024[134]). EYP-1901 (Duravyu), a long-acting formulation assessed in early-phase (Phase I and II) clinical evaluations for diabetic macular edema and neovascular age-related macular degeneration, was also highlighted in recent clinical investigations. It maintained consistent therapeutic benefits despite drastically lowering the frequency of dosage with the Durasert delivery system (Sayed et al., 2024[118]). All things considered, developments in topical drug delivery systems, especially those that use nanotechnology and formulations based on curcumin, provide a revolutionary way to treat a wide variety of skin disorders, enhancing clinical results, patient adherence, and therapeutic efficacy.

## Methodology

Under this review a thorough and methodical search of the literature was conducted in order to gather and examine pertinent scientific data on transdermal drug delivery methods, with an particular attention on formulations including curcumin and improvements based on nanotechnology. Databases including PubMed, Scopus, ScienceDirect, Google Scholar, and ClinicalTrials.gov were explored via keywords such as “*topical drug delivery*,” “*nanocarriers*,” “*nanogels*,” “*emulgel*,” “*transdermal patches*,” “*curcumin in dermatology*,” and “*skin penetration enhancement*.” Peer-reviewed papers from 2010 to 2024 were taken into account to make sure that both current developments and historical background were included. Commercial trends and clinical trial advancements were highlighted using additional data from regulatory databases, patents, and market reports. The selection of articles was focused on novelty, scientific rigor, and relevance; experimental research, clinical trials, meta-analyses, and high-impact review papers were given preference. The collected data underwent thorough analysis, was grouped according to formulation type and application area, and was then combined to provide a current and cohesive summary of the field's advancements and problems.

## Topical Drug Delivery Systems

The drug delivery system via skin represents a crucial therapeutic approach that offers numerous advantages over systemic administration, comprising minimized gastrointestinal irritation, circumvention of first-pass hepatic metabolism, and offering direct drug delivery to target sites while minimizing unnecessary adverse reactions (Zhao et al., 2024[149]). Despite its advantages, the skin's robust barrier nature, primarily due to the stratum corneum, presents a substantial challenge and therapeutic efficacy. This comprehensive review examines the various Drug delivery systems have been developed to overcome these barriers and improve the efficacy of topical drug administration. The skin poses a significant obstacle to drug penetration, largely due to the stratum corneum, the outermost layer of the epidermis. Drug molecules can penetrate the skin through three main pathways: transcellular (through skin cells), intercellular (between skin cells), & via skin appendages. Numerous factors influence drug penetration, including the physicochemical properties of the medication, the administration method, and the physiological condition of the skin. Percutaneous penetration varies significantly across different anatomical locations. Generally, the head, neck, and genitalia show the greatest absorption rates, while the trunk, back, and thighs demonstrate lower absorption rates. This regional variability must be considered when designing topical formulations and selecting application sites (Yuan et al., 2023[147]).

Traditional topical formulations i.e lotions, ointments, creams, lotions, & gels. These traditional dosage forms have been the mainstay of topical therapy but associated with several shortcomings consisting poor skin penetration, limited bioavailability, & lack of targeted delivery. Gels, because of their hydrophilicity, facilitate drug permeation through increased stratum corneum hydration (Paiva-Santos et al., 2023[92]). Conventional topical therapies frequently have limited efficacy due to the skin's barrier qualities prevent active chemicals from penetrating, decreasing drug delivery and therapeutic results. These systems may also have problems like low stability, limited drug-loading capacity, and skin irritation risk.

### Nanoparticle-based emerging topical drug delivery technologies

**Figure 2:**
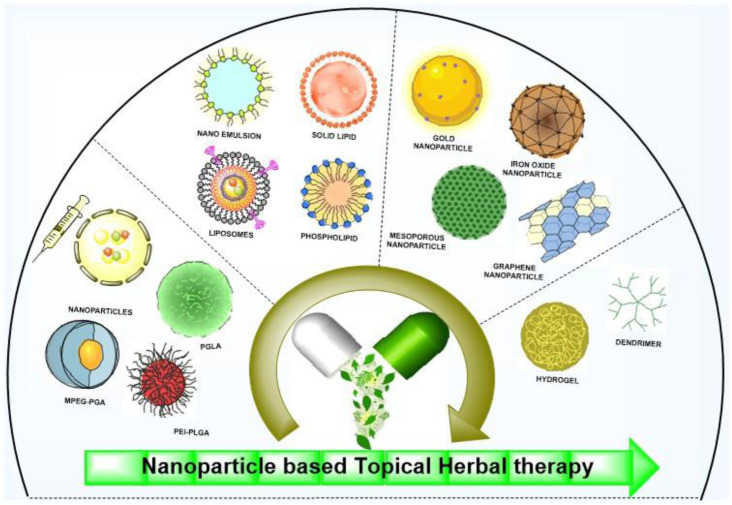
Nanocarrier-based herbal delivery systems for topical delivery of curcumin

Nanoparticle-based drug delivery approaches have been a game-changer in topical therapies, providing a number of benefits over traditional formulations. These nanoscale carriers have the potential to efficiently pass through the stratum corneum & transport active pharmaceutical ingredients (APIs) to specific dermal layers with regulated release patterns and improved bioavailability. Their broad applicability in dermatological therapy is attributed to their ability to encapsulate both hydrophilic and lipophilic drugs. These systems include polymeric nanoparticles, metal-based nanocarriers, solid lipid nanoparticles (SLNs), and nanostructured lipid carriers (NLCs) are some of the major studied nanoparticulate systems, shown in (see Figure 2(Fig. 2)). Each of these systems has distinct physicochemical characteristics that are suited for better skin retention, less irritation, and enhanced therapeutic efficacy. By resolving the basic issues with skin barrier penetration and targeted medication administration, nanocarrier-based delivery methods have transformed topical medicine. There are many difficulties because of the skin's superior barrier qualities, especially the stratum corneum which present significant challenges for effective drug penetration and therapeutic efficacy. Conventional topical treatments often exhibit limited efficacy because the skin's strong barrier function restricts the penetration of active ingredients, thereby reducing effective drug delivery and therapeutic outcomes (Yadav et al., 2025[145]), making them promising alternatives to overcome these barriers. Nanocarriers for topical delivery are as follows:

#### Lipid-based nanocarriers

Lipid-based nanosystems have shown improved performance in topical formulations by enhancing the intraocular half-life and bioavailability of therapeutic agents such as proteins, peptides, and genetic material (Miri et al., 2023[86]). As next-generation core-shell systems, lipid-polymer hybrid nanoparticles (LPHNPs) combine the advantages of polymeric nanoparticles with liposomes (Navarro-Partida et al., 2021[89]).

##### Lipid-based nanoparticles

Solid lipid nanoparticles have been identified as promising lipid-based dermal drug delivery platforms. SLNs offer better stability, regulated release, and epidermal penetration, although the mechanisms driving topical absorption and cellular uptake require further investigation (Liu et al., 2020[78]).

Lipidic nanocarriers known as nanostructured lipid carriers (NLCs) restore the capacity and permanence of therapeutic payloads. Because of their repeatable production processes and biocompatible lipidic excipients, NLCs-second-generation lipid nanocarriers with an unstructured matrix-offer potentially beneficial nanocarrier systems with marketable chances. These NLCs are currently acknowledged as a highly promising nanocarrier form for the effective delivery of medications through various modes of administration. To get beyond different ocular obstacles, biocompatible and biodegradable lipid nanoparticles have become a viable substitute for traditional ocular drug delivery methods. Over the past few decades of ocular therapy, lipid-based nanocarrier systems have produced significant technological breakthroughs and therapeutic benefits, including increased precorneal residence time, sustained drug release profile, minimal dosing frequency, reduced drug toxicity, targeted site delivery, and ultimately improved ocular bioavailability (Jacob et al., 2022[60]).

##### Cubosomes

Lipid-based lyotropic liquid crystals possess a highly ordered and thermodynamically stable internal nanostructure, making them promising matrices for sustained drug release. Cubosomes, which are nanoparticulate dispersion systems with great biocompatibility and bioadhesiveness, are created when cubic lipid phases are emulsified in water. The unique microstructure of cubosomes allows for the regulated release of active substances, improves drug stability, lowers toxicity, increases drug bioavailability, and boosts drug penetration after topical application (Gupta, 2023[52]).

##### Ethosomes

Ethosomes are vesicular nanocarriers based on lipids that have a high alcohol content. They have been designed to allow cutaneous and transdermal distribution of medicines with different physicochemical features. Since its inception in 1996, substantial research has concentrated on changing the original formulation through the insertion of various additional components, resulting in the development of multiple advanced ethosomal system variants (Abubakr et al., 2023[1]).

##### Self-Nanoemulsifying Drug Delivery Systems (SNEDDS)

Self-nanoemulsifying formulations represent an exceptionally promising approach for enhancing curcumin's topical bioavailability and therapeutic efficacy in addressing multiple skin disorders because of their unique capability to tackle the fundamental shortcomings linked with conventional curcumin formulations (Annisa et al., 2023[16]). SNEDDS are isotropic blends of oil, co-surfactant, and surfactant that spontaneously produce particle-sized nanoemulsions typically ranging from 10-200 nm when they come into contact with aqueous media, thereby significantly enhancing the solubility & skin permeability of lipophilic compounds like curcumin.

#### Polymeric nanocarriers

##### Polymer based nanoparticles

The stratum corneum (SC), the skin's outermost layer, presents a significant obstacle to successful penetration, despite the topical route being thought to be the most appropriate method for the targeted administration of medications to skin tissues for the treatment of localized dermatological problems. Drug molecules can be encapsulated in the core of polymeric nanoparticles or adsorbed onto their surface to greatly improve their physicochemical stability, prevent premature degradation, and allow for better permeation across the SC, increased retention within the skin layers, and modulation of release kinetics (Madawi et al., 2023[81]). Polycaprolactone and hyaluronic acid were used as the polymeric components in the solvent emulsification method for producing polymeric nanoparticles. The improved formulation showed an entrapment effectiveness of 83.33 ± 1.12 %, a mean particle size of 317.2 ± 1.27 nm, and a zeta potential of -0.25 ± 0.90 mV. A sustained release profile was shown by in vitro drug release tests, where 88.52 ± 1.10 % of the encapsulated medication was released over a 14-day period (Ahlawat et al., 2023[5]).

##### Hybrid lipid-polymer nanoparticles

NFX-loaded lipid-polymer hybrid nanoparticles have been developed with Eudragit RL100 as the polymeric component and Precirol ATO as the lipid matrix. Particle sizes in the produced formulations ranged from 28.92 to 730.30 nm, with a consistent and narrow size distribution. The zeta potential values of the nanoparticles, which ranged from +3.91 to +60.2 mV, indicated that their surface was positively charged. The optimized formulation achieved a satisfactory correlation between predicted and experimental results with a particle size of 159 nm, drug release of 92.61 %, and entrapment effectiveness of 79.2 % (Mohammed et al., 2025[87]).

##### Nanosponges

Three-dimensional, nanoscale porous structures called nanosponges can encapsulate a variety of therapeutic compounds and allow for targeted and regulated medication release. According to Ankem et al. (2023[15]) these carriers are capable of moving throughout the body, interacting with particular target areas, and releasing the loaded medicine in a predictable way. For controlled topical medication distribution, nanosponge technology has become a cutting-edge strategy. According to recent study, cyclodextrin-diphenyl carbonate nanosponges were made by convection heating and then freeze-dried to load luliconazole. The formulation demonstrated a high encapsulation efficiency (90.12 ± 0.92 %), indicating that the inclusion complex formation was successful. For the luliconazole-loaded nanosponges, laser light scattering analysis showed a limited, unimodal particle size distribution between 60 and 73 nm (Anusha and Mothilal, 2024[18]).

##### Nanocrystals

Nanocrystals are crystalline particles of a substance, usually between 1 and 1000nm long, which are almost entirely made of pure drug and contain no carrier material and which have an ordered molecular structure. They are mainly employed in pharmaceutical purposes to increase the solubility and dissolution rate of the poorly water-soluble drugs because of their high surface area that consequently increase the bioavailability and therapeutic effect. Top-down techniques (milling and high-pressure homogenization) and bottom-up techniques (precipitation) allow nanocrystals to be made, and so nanocrystals are an effective and versatile approach in the current drug delivery systems. In an attempt to enhance the delivery of curcumin to cells of the hepatocellular carcinoma (HCC), cellulose nanocrystals (CNCs) were utilized by Li et al. (2025[75]) to develop a pH-responsive nanocarrier. To create the system, CNCs were coated with polydopamine (PDA), then 3-carboxyphenylboronic acid (CA) was grafted onto the coat to be able to target the system. Curcumin was later encapsulated through boronate ester conjugation with the drug loading efficiency of 10.58 +1.32. It showed high pH-sensitive release, and the relative drug release was increased to the acidic environment (pH 5.4) by a factor of 3.18 than that under physiological pH (7.2) environment, which resembled the tumor microenvironment. Moreover, the CA-functionalized system promoted improved cellular uptake and targeting of HCC cells, which led to higher intracellular drug concentrations and better cytotoxicity in two-dimensional, as well as, three-dimensional in vitro systems. In general, this research indicates the potential of CNC-based, boronic acid functionalized nanocarriers in targeted and stimuli-responsive delivery of poorly water-soluble anticancer agents (Li et al., 2026[76]).

##### Bacterial nanocellulose (BNC)

Bacterial nanocellulose membranes demonstrate significant potential for topical drug delivery owing to their intrinsic biocompatibility & distinct three-dimensional nanoporous framework. BNC systems show excellent storage stability under various conditions and good cutaneous compatibility, making them promising dermal delivery systems (Silva et al., 2020[126]).

#### Inorganic nanocarriers

##### Mesoporous silica nanoparticles (MSNs)

Mesoporous silica nanoparticles (MSN) have been formulated for topical application of hydrophobic drugs. These systems show enhanced drug release rates and improved antioxidant activity compared to conventional formulations. MSN demonstrates biocompatibility and chemical/thermal stability (Pawar et al., 2021[99]). These inorganic nanocarriers allow for drug delivery via a variety of administration methods and have flexible physicochemical characteristics. Lipid-based surface modification has been demonstrated to lessen the first burst release, especially for water-soluble medications. Adding oleic acid (OA) as a lipid component improves skin penetration even further. Interestingly, the method showed a sustained drug release profile over 48 hours and high encapsulation efficiency (97.6 ± 1.8 %) (Slavkova et al., 2024[128]). In comparison to the hydrogel containing free medication, the in vitro study permeability demonstrated a 2.35-fold improvement. The MSN were discovered to be chemically and thermally stable, biocompatible nanoparticles (Djayanti et al., 2023[42]).

#### Metallic nanoparticles

##### Gold nanoparticles

The distribution of the hyaluronic acid-coated gold nanoparticles in the eyes of mice was compared to that of topically applied uncoated gold nanoparticles and phosphate-buffered saline (PBS). Hyaluronic acid-coated gold nanoparticles acquired much larger dispersion in the posterior portion of the eye compared to uncoated gold nanoparticles, according to results from all characterization techniques used in this work (Labala et al., 2015[71]). Gold nanoparticles coated with layer-by-layer (LbL) polymers showed an average particle size of 98.5 ± 4.3 nm and a zeta potential of 32.3 ± 1.3 mV. The loading efficiency of IM in LbL-AuNPs was 28.3 ± 2.3 %, which is the highest loading of a small-molecule medicine in gold nanoparticles that has been reported. Iontophoretic application increased the skin penetration of IM-loaded AuNPs by 6.2 times as compared to passive application, according to in vitro skin penetration tests employing excised porcine ear skin (Laradji et al., 2021[72]).

##### Silver nanoparticles

Silver nanoparticles (AgNp) of different sizes, forms, and structures can be produced by modifying the synthesis process; these variations have a significant effect on the biological effect, particularly the control of antibacterial activity. It was discovered that the biosynthesized nanoparticles were spherical, homogeneous, stable, and ranged in size from 9.21 nm to 14.03 nm (Velescu et al., 2023[142]). When compared to the crude form of the plant extract and latex, the gel containing nanoparticles demonstrated excellent antibacterial efficacy against *Escherichia coli*, *Bacillus subtilis*, and *Staphylococcus aureus*. AgNP-containing PVA/PEG films showed strong antibacterial activity against both Gram-positive and Gram-negative bacterial strains. While povidone iodine only stops the development of some bacteria, anisotropic AgNPs can stop the growth of the majority of tested bacterial pathogens and offer protection for more than 48 hours (Rolim et al., 2019[114]).

#### Others

##### Dendrimers

Poly(amidoamine) dendrimers have been extensively studied as possible nanomaterials that could improve topically administered medications' skin penetration. Numerous studies have shown that adding dendrimers to a topically applied formulation can greatly increase the amount of medicine entering and passing through the skin under specific conditions and for specific medications. A dendrimer based on a well-defined branching structure for nanomedical devices with a well-defined nanoarchitecture was grafted using a self-assembly technique, producing spherical, very homogeneous molecules with several surface functions (Huang et al., 2015[57]). A self-assembling dendrimer-conjugated method has been developed to distribute isotretinoin (13-cis-retinoic acid, 13cRA-D) transdermally. The technology demonstrated a controlled-release profile, which is defined by a gradual release of the medication in physiologically normal settings and an accelerated release in low-pH situations like inflamed tissues. In a related strategy, a unique transdermal drug-delivery system developed to improve diclofenac's skin penetration by mixing sonophoresis with a polyamidoamine (PAMAM) dendrimer. After 24 hours, the diclofenac-dendrimer gel without sonophoresis achieved a substantially higher cumulative penetration of 257.3 µg/cm², while the diclofenac gel without dendrimer and ultrasonic therapy produced a cumulative permeation of 56.69 µg/cm² (Kirkby et al., 2024[68]).

##### Nanosuspensions

Nanosuspensions have gained attention as a strategy for improving bioavailability of hydrophobic drugs showing limited solubility. These systems show increased skin adhesiveness, enhanced saturation solubility and dissolution rate, thereby improving cutaneous distribution. Nanosuspensions are particularly beneficial for active ingredients with low to medium solubility (Aldeeb et al., 2024[11]).

#### Vesicular drug delivery systems

##### Transfersomes and niosomes

Vesicular systems such as transfersomes & niosomes have been incorporated within hydrogel formulations for enhanced topical delivery. These systems enable controlled drug delivery, enhance skin penetration, & reduced drug toxicity in comparison with traditional dosage forms. Studies have shown that these vesicular drug delivery systems do not significantly affect the rheological properties of hydrogel matrices (Cristiano et al., 2020[35]).

##### Bioadhesive nanoparticles

Bioadhesive nanoparticles (BNPs) perform dual functions *i.e* bioadhesives & nanocarriers, mediating targeted drug delivery system, prolonging retention time, & enhancing drug penetration via skin layers. These systems have a number of benefits, such as improved adhesive strength, flexibility, biocompatibility, and biodegradability. Biopolymeric nanoparticles (BNPs) have showed effectiveness in the treatment of numerous skin problems, such as atopic dermatitis, skin cancer, psoriasis, & microbial infections (Almuqbil and Aldhubiab, 2025[13]).

### Advanced physical enhancement techniques

#### Microneedle technology

##### Dissolving microneedles

Dissolving microneedles (DMN) represent a revolutionary approach for painless & direct dermal drug delivery system. These hydrophilic, polymer-based constructions enable penetration across the stratum corneum to enhance drug delivery while providing minimal discomfort. Clinical evidence supports their effectiveness in treatment of several skin conditions such as aging, hyperpigmentation, psoriasis, warts, & keloids (De Decker et al., 2023[39]).

##### Hollow microneedles

Hollow microneedles provide transformative solutions for topical diagnosis & therapeutic applications. 3D printing technology has advanced the fabrication of these devices, enabling high-resolution manufacturing with sophisticated features. These systems provide adjustable dosing capabilities and integration with microfluidic devices (Ghaznavi et al., 2025[48]).

##### Iontophoresis

Iontophoresis represents a well-established technique that increases drug penetration through the skin by using a low-intensity electrical current to promote the transdermal absorption of ionized substances. Recent developments include portable electric facial care devices that can effectively deliver various cosmetic and therapeutic compounds (Touma et al., 2023[136]).

##### 3D printing method

Topical formulations with accurate drug dosage and individualized release patterns can be made with 3D printing. This technology offers opportunities for patient-specific therapy optimization.

### Specialized delivery systems

#### Hydrogel-based systems

Hydrogel-based delivery systems have gained considerable attention as promising approaches for topical therapy. These systems enable high drug-loading efficiency with controlled release, enhanced skin barrier penetration, and greater biocompatibility is made possible by these methods. Stimuli-responsive hydrogels are being developed for enhanced targeting and responsiveness (Rouf et al., 2024[116]).

##### Microemulsion systems

Microemulsion formulations provide enhanced drug solubility and penetration. These systems can be formulated as cream-gel or gel-in-oil emulsions to optimize drug penetration profiles while maintaining suitable rheological properties for application. Nanocarrier designs vary significantly, providing controlled drug release and enhanced skin penetration.

Topical drug delivery systems have demonstrated effectiveness in treating various skin disorders comprising microbial infections, skin cancer, dermatitis, burn injuries, wounds, & psoriasis. Novel delivery systems have shown promise in managing chronic inflammatory conditions where conventional treatments have limited efficacy (Gavinet et al., 2024[46]).

### Mechanisms of enhanced skin penetration

#### Physical enhancement mechanisms

Nanocarriers enhance skin penetration through various mechanisms including occlusion and changes to surface tension. The outermost non-viable layer of the epidermis, the stratum corneum, is a significant barrier to the passage of drugs through the skin. To get over this restriction and improve skin penetration, a variety of nanocarrier technologies have been developed as drug-delivery vehicles (Dhule and Nandgude, 2023[41]).

##### Size-dependent penetration

Conventional eye drops, which deliver less than 5 % of the administered medicine to ocular tissues, make up the majority of commercially available ophthalmic formulations. Many physiological barriers, such as the corneal and conjunctival epithelia, lacrimal drainage, quick tear turnover, the blood-retinal barrier, enzymatic drug degradation, and reflex blinking, are the main causes of the limited ocular bioavailability (Akhter et al., 2022[8]).

### Clinical applications 

#### Dermatological conditions

Nanotechnology-based treatments have shown great promise in the treatment of skin cancer by facilitating more efficient medication delivery. However, inadequate medication penetration into the stratum corneum or tumor lesions, low therapeutic efficacy, and the requirement for higher concentrations of active pharmaceutical ingredients to produce the intended impact are frequently the limitations of conventional treatments (Akhter et al., 2022[8]).

##### Inflammatory skin diseases

Conventional topical treatments for inflammatory skin diseases are often limited by poor skin penetration and long-term adverse effects. Au-MEA nanoparticles (NPs), on the other hand, showed better stability and increased penetration when compared to traditional Au-CA NPs. *In vivo*, transcutaneous delivery of Au-MEA NPs produced strong therapeutic effects against psoriasiform and rosacea-like dermatitis without any evident adverse reactions (Yadav et al., 2025[145]).

##### Wound healing and antimicrobial applications

Polyvinylpyrrolidone (PVP) and chitosan are examples of synthetic and natural polymeric micro- and nanocarriers that are essential for the healing of infected chronic wounds. Growth factors (GFs) and metal or metal oxide nanoparticles (MNPs/MONPs) are two promising approaches to improve different stages of wound healing, such as hemostasis, inflammation, proliferation, and remodeling/maturation, and to help eradicate bacterial and fungal infections (Zhao et al., 2024[148]). In context to this some recent studies have reported the utilization of these polymers such as Hu et al. (2026[55]) designed a multifunctional hybrid hydrogel scaffold (SF@NP) through microfluidic 3D printing in the treatment of infected diabetic wounds. The scaffold was also loaded with copper/iron bimetallic nanoparticles (NP-Cur) which were loaded with curcumin and which displayed enzyme-like (oxidase-and peroxidase-like) activity in reactive oxygen species (ROS)-scavenging. There is also the fact that the system was shown to have a multimodal antibacterial response due to the release of copper ions and photothermal activity. The discharged NP-Cur stimulated fibroblast migration, collagen formation and angiogenesis, which enhanced regeneration of the tissue. Notably, curcumin enhanced immunomodulation by changing the macrophage polarization of the pro-inflammatory M1 to the regenerative M 2. The scaffold efficiently reprogrammed the wound microenvironment *in vivo*, with a reduction in oxidative stress, suppression of inflammation, neovascularization, and M2 macrophage increase. Generally, this work has demonstrated that there is a potential of an immunomodulatory hydrogel platform in enhancing healing of chronic wounds and diabetic tissue healing.

**Antifungal applications: **Silver nanoparticle-loaded oregano oil nano emulsion exhibited enhanced antimicrobial activity, as indicated by larger zones of inhibition compared to the standard ketoconazole and oregano oil combination. This novel formulation holds great promise for the treatment of Mucocutaneous candidiasis, providing an effective and potentially safer alternative to current therapies (Alavi and Rai, 2020[10]).

### Advantages of nanocarrier systems

Targeted medication delivery, flavor masking, decreased adverse effects, enhanced water solubility, adjustable particle size, and ease of commercial production are some benefits of these nanocarrier systems. They also show little cytotoxicity, stability, and biocompatibility. These technologies improve therapeutic efficacy at a given dose by delivering the medication directly to a specific target spot instead of dispersing it systemically (Sonker et al., 2023[129]).

#### Limitations and challenges

Disadvantages include limited encapsulation for larger molecules. The drug-dendrimer interaction and the selection of a particular dendrimer are crucial for attaining the best enhancement, as evidenced by the fact that in certain instances, dendrimers offered little to no improvement in skin penetration. Furthermore, the majority of research to date has been done *in vitro*, with little progress made beyond laboratory investigations.

### Future perspectives and emerging trends

**Smart and responsive systems: **The development of stimuli-responsive systems and smart drug delivery technologies represents a major trend. These systems exhibit responsiveness to specific biological or environmental triggers for enhanced targeting and controlled release.

**Personalized medicine strategies: **Future trends in topical drug delivery focus on personalized medicine approaches utilizing biomarkers and multi-omics technologies. These approaches aid in diagnosis, predicting treatment responses, & informing treatment decisions.

**Nanotechnology integration: **The cosmetic and pharmaceutical industries are increasingly incorporating nanotechnology for enhanced skin permeation, enhanced bioavailability, prolonged efficacy, decreased toxicity, & superior stability. Nano cosmetics represent a growing market with applications in precision medicine (Alfiya et al., 2024[12]).

## Skin Diseases: Prevalence, Causes, and Pathophysiology

### Inflammatory skin disorders

#### Psoriasis: the symptoms, causes and treatments

Psoriasis is a persistent inflammatory dermatosis characterized excessive proliferation of epidermal cells, causing thickened, inflamed areas adorned with glistening scales. This disease is recognized as one of the most prevalent conditions stemming from immune system dysfunction, with global prevalence rates ranging from 0.6 % to 4.8 %. Psoriasis develops by an intricate relationship between environmental variables alongside genetic predisposition and triggers, leading to an intensified immune response mostly characterized by T helper 1 cytokines, such as TNF-α. This results in an immune system imbalance, causing the over proliferation of skin cells, known as keratinocytes, and the development of plaque psoriasis (Podobińska, 2025[106]).

Psoriasis symptoms may manifest at varying degrees of severity, with some being more advanced than others. Typically, the symptoms present as

- Erythematous plaques adorned with silvery scales
- Dehydrated and fissured skin with a potential for haemorrhaging
- A pruritic or burning sensation
- Thickened or pitted nails
- Swollen and rigid joints (in instances of psoriatic arthritis).

In the absence of discomfort, the present approach to psoriasis treatment aims to ameliorate patch inflammation and decelerate skin cell renewal. This is accomplished using creams including topical steroids, vitamin D ointment, phototherapy utilizing UVB light, and other potent medications such as methotrexate and targeted biologic therapy. Treatment with TNF inhibitors has proven effective for moderate to severe psoriasis by targeting regions delineated by inflammation and psoriatic lesions.

##### Eczema (atopic dermatitis): etiology, clinical features, and treatment

A prevalent kind of eczema, atopic dermatitis is a chronic inflammatory disease characterized by dry, pruritic skin, with scratching leading to eczematous lesions. The etiology of atopic dermatitis is multifaceted, with significant elderly factors including genetics, environmental influences, and immune system dysfunction. Individuals afflicted with atopic dermatitis exhibit a compromised skin barrier due to mutations in the filaggrin gene, hence elevating the *trans* epidermal water loss (TEWL) rate and susceptibility to detrimental agents (Brănișteanu et al., 2025[30]).

**The manifestations of eczema encompass:**

- Severe pruritus, potentially exacerbated during nocturnal hours
- Skin exhibiting areas of red or brownish-gray pigmentation
- Scaly or hyperkeratotic integument
- Small, elevated lesions that may exude fluid upon abrasion.

The management of atopic dermatitis focuses on repairing the skin barrier and regulating inflammation. Topical corticosteroids are efficacious in managing inflammation and pruritus. In more severe instances, inflammation management, emollient application, and flare prevention become essential alongside enhanced skin hydration. Alternative immunosuppressive systemic methods or biologics, such as dupilumab, may be employed to manage more severe symptoms. Comprehending the pathophysiology is crucial for developing significant treatment methods for both psoriasis and eczema, as they delineate distinct health indicators that compromise public health (Costa et al., 2022[34]).

### Infectious skin diseases

Dermatological conditions caused by infectious agents, such as germs, viruses, and bacteria, adversely affect the patient, resulting in disability and diminished quality of life. This is a summary of the prevalent observations of skin infections categorized by their infectious agents (Billings, 2020[27]).

#### Bacterial infections

**Acne vulgaris:** Acne, the most prevalent type of skin condition, is defined by a persistent inflammatory process mostly impacting the sebaceous glands. Acne is featured by the presence of comedones, papules, pustules, &, in certain cases, cystic forms. The primary causal component is the proliferation of Cutibacterium acnes, resulting in irritation and obstruction of hair follicles. Current treatment protocols encompass topical therapy utilizing retinoids, cleansing with benzoyl peroxide, and in situations of extreme severity, the administration of systemic antibiotics (Sutaria et al., 2023[132]).

**Impetigo:** A highly contagious bacterial dermatological condition predominantly affecting school-aged children caused by either *Staphylococcus aureus* or *Streptococcus* pyogenes. It manifests as red lesions that rupture, exude fluid, & frequently produce a yellow crust. Severe infections are typically managed with oral antibiotics, as well as topical agents such as mupirocin (Hartman-Adams et al., 2014[54]).

**Folliculitis:** This condition refers to the inflammation of hair follicles resulting from infection, irritation, or obstruction. Numerous bacterial species, including *Staphylococcus aureus*, can contribute to this illness. It presents as one or more little pink papules or pustules surrounding the hair, characterized by red, swollen regions, occasionally with pus on the surface. It is frequently managed with topical antiseptics or antibacterial drugs (Kashikar et al., 2024[63]).

##### Mycological diseases 

**Fungal infections:** Candidiasis, characterized by the uncontrolled proliferation of *Candida* species (most commonly *C. albicans*), can affect various body regions, including the skin and oral thrush. This illness, marked by red, itchy rashes and potential white patches, is treatable with antifungal medications like fluconazole or clotrimazole (Brown et al., 2024[31]).

**Ringworm:** Dermatophytosis is a fungal infection caused by Trichophyton, Microsporum, and Epidermophyton, resulting in circular and elevated rashes on the skin. Treatment primarily involves topical antifungal medications, such as terbinafine for mild infections, or systemic antifungals for more severe cases (Kamasani et al., 2024[62]).

##### Viral pathologies

**Herpes simplex virus (HSV):** HSV-1 induces oral herpes, while HSV-2 targets the genital area, resulting in genital herpes. Lesions or vesicles will manifest on the affected region. No proven cure for herpes exists; however, outbreak management can be achieved with the use of acyclovir pills, in conjunction with measures to reduce transmission risk (Piperi et al., 2024[104]).

**Warts:** Induced by the human papillomavirus, they are elevated skin lesions that can develop on any part of the body. Treatment options include the use of salicylic acid, laser therapy for older lesions, and cryosurgery (Jabłońska et al., 1997[58]).

### Skin pigmentation disorders

Skin pigmentation disorders are classified into two primary categories: hyperpigmentation and hypopigmentation, each characterized by distinct clinical characteristics and underlying causes. This is a summary of prevalent conditions within these categories.

#### Hyperpigmentation

This syndrome occurs when extra melanin is produced in specific parts of the body, resulting in those spots being darker than the surrounding skin (Pérez-Bernal et al., 2000[101]). Two significant categories comprise:

##### Melasma

This disorder primarily affects areas of the face that are exposed to the sun and appears as symmetrical brown to gray-brown patches. It is predominantly induced by ultraviolet radiation, hormonal fluctuations, such as those occurring during pregnancy, and genetic predisposition. The fundamental pathophysiology entails heightened melanocyte activity resulting from the influence of estrogen and ultraviolet radiation. Treatment modalities comprise topical medications (hydroquinone and retinoids), chemical peels, and laser therapy to diminish pigmentation (Ghasemiyeh et al., 2024[47]).

##### Post-inflammatory hyperpigmentation (PIH)

This transpires because of an injury or inflammatory condition of the skin, i.e acne, eczema, or psoriasis. The alteration of melanin results in pigmentation due to the excessive synthesis of melanin during the healing process. This may be more pronounced in those with darker skin tones. Management options encompass topical treatments that promote skin turnover, such as alpha hydroxy acids, and the application of sunscreens to avert more darkening (Mar et al., 2024[83]).

##### Hypopigmentation

Decreases in melanin production are termed hypopigmentation, resulting in lighter skin regions. Significant stipulations encompass: 

##### Vitiligo

An autoimmune disorder marked by the loss of melanocytes, giving rise to depigmented spots on the skin. The etiology remains ambiguous; however it is believed to pertain to genetic factors and immune system dysfunction. Any bodily region may be impacted and could deteriorate with time. Treatment encompasses topical corticosteroids, phototherapy, and, in extreme cases, depigmentation medications (Akl et al., 2024[9]).

##### Albinism

A genetic condition characterized by a full or partial deficiency of melanin in the skin, hair, & eyes, causing from certain gene mutations, such as OCA2. They are at an elevated risk of sunburn and skin malignancies throughout their lifetime due to insufficient protective pigmentation. Its management encompasses appropriate sun exposure recommendations and addressing additional health concerns such as eye impairments (Anotida et al., 2024[17]).

Hypopigmentation and hyperpigmentation problems can considerably impact an individual's psychological condition & overall well-being. To design specialized treatments, it is essential to identify the causations. These encompass topical treatments and advanced interventions such as laser therapy, designed to restore natural skin pigmentation and enhance skin health (Gao and Xiang, 2025[45]).

### Skin cancer and precancerous lesions

Skin cancer is among the rapidly proliferating health disorders, classified by its various forms and cellular origins. According to the previous definition, the three predominant kinds of skin cancer are melanoma, basal cell carcinoma (BCC), & squamous cell carcinoma (SCC) (Valian et al., 2024[141]). Each category possesses distinct risk factors and treatment modalities.

#### Melanoma

This type of skin cancer is the most life-threatening, originating as a mole that may evolve over time into a significant threat. This particular type originates from melanocytes, the cells responsible for producing skin pigment. Melanoma, owing to its extreme peril, has the potential to metastasize to other areas of the body. It typically manifests as a completely new mole or an existing one that alters in characteristics, exhibiting asymmetry, an uneven border, numerous colours, and a diameter exceeding six millimetres. Early-stage treatment has demonstrated favourable outcomes (Duncan, 2009[43]).

##### Basal cell carcinoma (BCC)

The most prevalent kind of skin cancer, basal cell carcinoma (BCC), develops from the epidermis' basal cells. This type of skin cancer may manifest as a lump that is pearlescent or waxy in appearance. This condition manifests on areas of the skin that have been subjected to sunlight exposure. The likelihood of BCC metastasizing is minimal but, if left untreated, it can cause significant tissue damage. Surgical excision, Mohs micrographic surgery, and topical therapy represent some viable treatment modalities (Kasumagic-Halilovic et al., 2019[65]).

##### Squamous cell carcinoma (SCC)

**Figure 3:**
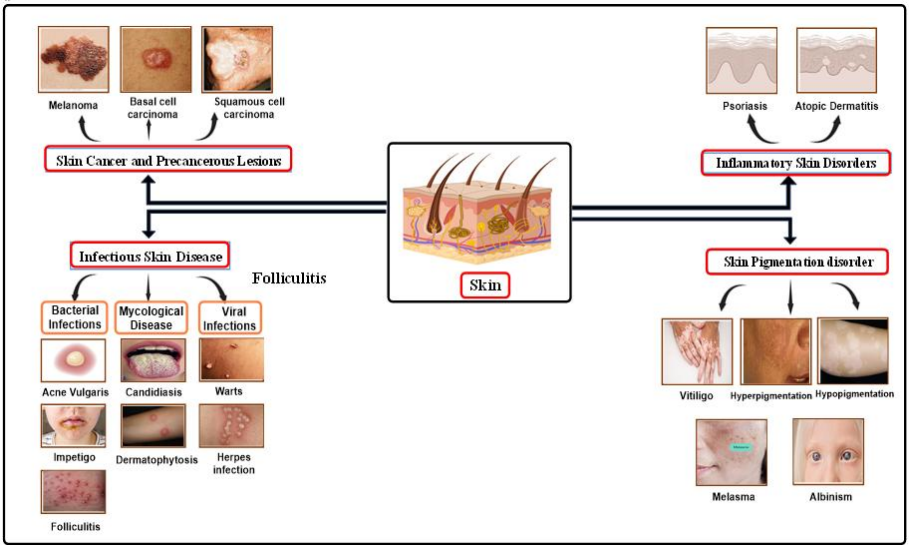
Curcumin-loaded liposomes facilitating targeted and sustained skin delivery

SCC develops from the squamous cells of the epidermis and may present as a scabbing sore, a scaly lesion, or a firm red nodule. Squamous cell carcinoma (SCC), similar to basal cell carcinoma (BCC), is linked to ultraviolet (UV) radiation exposure; however, SCC has a higher propensity for dissemination or metastasis than BCC. Treatment typically involves surgical excision, radiation, or topical chemotherapy (Cassarino et al., 2006[32]). The lipid-based nature of liposomes allows for effective incorporation of curcumin and favorable interaction with the stratum corneum (Figure 3(Fig. 3)).

### Factors of risk

**Ultraviolet (UV) radiation:** Prolonged exposure to UV radiation whether from natural sunlight or artificial sources i.e tanning beds, significantly elevates the risk of acquiring many forms of skin cancer. Studies demonstrate that the utilization of sunbeds is associated with a heightened risk of getting melanoma (D'Orazio et al., 2013[36]).

**Skin type:** Individuals with a fair complexion and light hair possess a heightened susceptibility to skin cancer due to diminished melanin levels, which offer insufficient protection against UV radiation. 

**Family history:** An individual's genetic susceptibility to skin cancer is heightened if there is a familial history of the disease; hence elevate the probability of developing skin cancer (Scherer and Kumar, 2010[119]).

**Age:** The likelihood of developing skin cancer occurrence escalates with age due to cumulative sun exposure over time, which exacerbates DNA damage in skin cells.

**Immune suppression:** Individuals with weakened immune systems, i.e organ transplant recipients, exhibit an increased vulnerability to skin malignancies (Paulson et al., 2019[98]).

## Herbal Remedies in Topical Therapeutics: A Focus on Curcumin

Herbal remedies have emerged as pivotal components in modern topical therapeutics, offering natural alternatives to synthetic pharmaceuticals with enhanced safety profiles and multifaceted therapeutic benefits (Madawi et al., 2023[81]) Among the vast array of bioactive phytochemicals available for topical applications, curcumin stands out as one of the most extensively studied and clinically promising compounds (Raghav et al., 2024[110]). Curcuma longa has been utilized for decades in both topical and oral treatments due to its low toxicity and health-promoting benefits. The turmeric rhizome contains the polyphenolic chemical curcumin, which is the plant's main bioactive component (Kasprzak-Drozd et al., 2024[64]) This comprehensive review explores the role of curcumin in topical drug delivery systems, examining its therapeutic mechanisms, formulation challenges, and clinical applications in dermatological conditions (Gunawardana and Dias, 2024[49]).

### Curcumin as a therapeutic agent for skin diseases

Curcumin monotherapy and combination therapy significantly improved Psoriasis Area and Severity Index (PASI) scores when compared to control groups, according to a thorough meta-analysis of 26 studies, including seven randomized clinical trials and 19 preclinical investigations. The analysis showed a standard mean difference of -0.83 % with 95 % confidence interval of −1.53 to 0.14 and p = 0.02. Preclinical evaluation showed that curcumin was more effective than controls at improving the phenotype of psoriatic dermatitis in mice, as shown by lower expression of inflammatory cytokines such as interleukin (IL)-17, IL-17F, IL-22, and tumor necrosis factor (TNF)-α, as well as lower total PASI scores (Józsa et al., 2023[61]). Chopra et al. (2021[33]) reviewed the therapeutic potential of *Curcuma longa* and its principal bioactive compound, curcumin, emphasizing its wide-ranging pharmacological activities including antibacterial, antiprotozoal, anticancer, antidiabetic, anti-obesity, and wound healing effects. The paper shows major shortcomings of curcumin including low bioavailability, poor solubility, and rapid metabolism, which make the use of curcumin in clinical settings limited. To overcome these obstacles, different nano formulation methods were being discussed, such as the methods to increase the solubility, the bioavailability, increase the duration of systemic circulation, and the target delivery. The authors also underlined the importance of nanotechnology in the control of physicochemical and biological characteristics of curcumin, which enhances its therapeutic effectiveness. Moreover, the review also draws a list of the latest developments and replicable formulation plans to make industrial production and community-wide implementation. On the whole, the research highlights the importance of nano-based delivery system to surpass the natural constraints of curcumin and increase its clinical applications.

Similarly, another study reported by Rao et al. (2024[113]), the anti-inflammatory, antioxidant, and antibacterial qualities of curcumin, a polyphenolic substance extracted from the rhizome of *Curcuma longa L.*, have been utilized for millennia. Their thorough analysis demonstrated that curcumin's antiviral, antimutagenic, and antifungal properties make it an excellent choice for the prevention and treatment of skin disorders like inflammation, psoriasis, acne, and early aging. The authors also showed that curcumin promotes collagen deposition, speeds up wound healing, and guards against skin damage brought on by extended UVB exposure. Curcumin has also been demonstrated to boost fibroblast and vascular density in wounds, indicating its promise as a low-cost, well-tolerated, and efficient treatment for a variety of skin conditions. Rao et al. (2024[113]) developed a Phyto-dermal gel (PDG) containing polymeric mixed micelles (PMMs), consisting of Pluronic F127 and Pluronic F68, to have both sun-protective and antioxidant properties. Curcumin and quercetin were co-loaded into PMMs and optimized with the 3 2 factorial design with the parameters of vesicle size, SPF, entrapment efficiency, and antioxidant potential compared. The optimized micelles had nanoscale sizes (387527 nm) and moderate polydispersity and spherical shape. The synergistic effect led to an increase in SPF (18.8628.32) and the antioxidation activity of the combination of phytoconstituents. They developed optimized PMMs and inserted them into a Carbopol 940 gel, which exhibited appropriate physicochemical characteristics such as, having the right pH, being spreadable, and thixotropic. The best formulation was one with an SPF of 27 + 0.5 and low syneresis at 30 days, and desirable *ex vivo* permeation and retention on the skin. Notably, there was absence of erythema or edema in the Wistar rats establishing its safety. On the whole, the current paper shows that nanocarriers comprising of PMMs are able to improve the dermal delivery and protective efficacy of phytoconstituents to be used in skin care.

Similarly, Koch et al. (2023[69]) highlighted the development of innovative topical and transdermal nanotechnology-based platforms to address the low therapeutic efficacy, safety issues, and patient noncompliance related to traditional dose forms. Their review focused on the latest developments in nanoemulgels for skin applications, which contain curcumin and other naturally derived substances for wound healing, skin and skin appendage infections, inflammatory skin illnesses, skin cancer, neuropathy, and anti-aging. According to the scientists, the proposed formulations outperformed currently available products in terms of droplet size, polydispersity index, viscosity, spreadability, pH, stability, drug release, and skin penetration. Additionally, these formulations' safety and therapeutic performance were validated by *in vitro* and *in vivo* experiments, underscoring their potential as viable substitutes or supplements to current treatments.

A comprehensive review of developments in hydrogel development and the application of curcumin for the treatment of chronic wounds was carried out by Patel et al. (2024[95]). Curcumin, a phytopharmaceutical with antioxidant, anti-inflammatory, and pro-angiogenic qualities, is helpful in boosting wound healing, according to their thorough analysis of 51 research published between 2006 and 2024. By controlling the wound surrounding tissue, enabling regulated release of active compounds, and offering spatiotemporal control during healing, the scientists showed that hydrogels offer substantial benefits in wound care. Additionally, their research revealed that curcumin-loaded hydrogels have potential pharmacological and cosmetic benefits in addition to treating inflammatory diseases, malignancies, liver disorders, asthma, and osteoarthritis. In order to improve psoriasis outcomes of treatment, Chen et al., (2024) produced an ionic liquid hydrogel based on curcumin that is loaded with ilomastat for topical transdermal delivery. They showed that local application of this hydrogel alleviated skin lesions and markedly decreased the production of inflammatory mediators, matrix metalloproteinase-8, and collagen-I using an imiquimod-induced psoriasis mice model. Additionally, ferroptosis-related proteins SLC7A11 and ASL4 were significantly reduced by treatment. Additionally, after treatment, certain bacterial populations in psoriatic mice returned to normal, according to gut microbiota research. These results imply that the ionic liquid hydrogel based on curcumin is a multipurpose, non-invasive, non-irritating, and very successful percutaneous treatment for psoriasis.

Curcumin, a polyphenolic molecule, has gained increasing focus for its potential application in the treatment of many skin disorders owing to its unique pharmacological properties. Curcumin is obtained from the rhizome of *Curcuma longa* and is recognized for their anti-inflammatory qualities, antioxidant processes, antibacterial effects, & wound healing capabilities. Curcumin modulates important inflammatory pathways to produce strong anti-inflammatory effects. Pro-inflammatory cytokines including TNF-α and IL-6, which are important in the pathophysiology of psoriasis and eczema, are suppressed by it. Current data indicates that curcumin can downregulate *cyclooxygenase*-2 (*COX-2*) and *lipoxygenase* (LOX), which are recognized as the enzymatic proteins of the inflammatory effector system. This effect is beneficial in managing dermatitis problems characterized by a persistent inflammatory component (Aggarwal and Harikumar, 2009[4]). Curcumin is recognized for its preventive efficacy against several skin pathogens, including bacteria, fungus, and viruses. Curcumin has been demonstrated to limit the proliferation of certain Gram-positive bacteria, including *Staphylococcus aureus*, which is linked with skin illnesses i.e acne and impetigo. Additionally, curcumin has demonstrated antifungal properties against dermatophytes and yeast, which are associated with illnesses like candidiasis. 

### Mechanisms of curcumin in skin disease treatment

**Figure 4:**
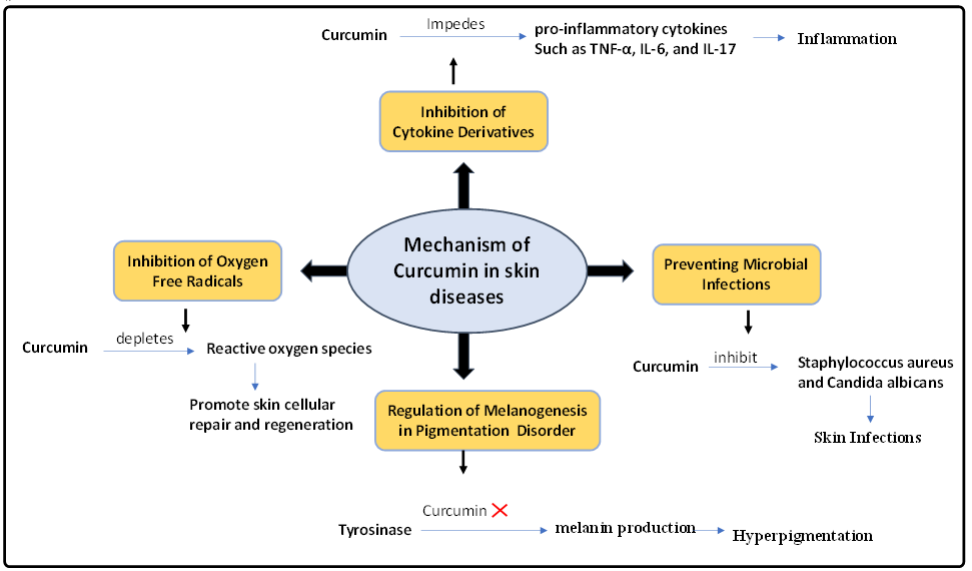
Mechanisms underlying curcumin's effects in the treatment of skin diseases

Curcumin, a bioactive constituent of turmeric with diverse medicinal attributes, contributes to the management of numerous skin disorders via multiple mechanisms. Curcumin inhibits microbial causal factors, regulates oxidative stress, modulates cytokines, and controls melanogenesis in pigmentation disorders. Curcumin is recognized for its ability to impede the progression of pro-inflammatory cytokines & inflammatory skin conditions, namely targeting TNF-α, IL-6, & IL-17 (Sun et al., 2013[130]). In psoriasis and eczema, inflammation is pivotal and indicative of advanced disease, therefore presenting a significant concern. Numerous literatures have confirmed that curcumin is found to be beneficial in modulating and downregulating inflammatory signalling pathways, including NF-κB and related inflammatory processes shown in Figure 4(Fig. 4) (Serini et al., 2025[120]).

Curcumin's efficacy can be assessed in relation to psoriasis by examining the quantity of pro-oxidants that curcumin can influence. Moreover, it depletes the driving enzyme, resulting in the overproduction of superoxide, hence facilitating contraception and generating reactive oxygen species (Peng et al., 2021[100]). When the quantities of specific oxidative species are diminished, the skin experiences several benefits, including cellular repair and regeneration, hence enhancing cellular protection against injury and promoting resilience. Curcumin also exhibits a significant antimicrobial effect against microorganisms responsible for skin infections, including bacteria, fungus, and viruses. It is recognized for its ability to inhibit *Staphylococcus aureus* and *Candida albicans*, which are prevalent causal agents of skin infections. Curcumin inhibits infections that may exacerbate the inflammatory processes of acne and dermatitis by damaging microbial cell membranes and preventing biofilm formation (Praditya et al., 2019[107]). Moreover, curcumin contributes to regulating melanin formation in the skin (melanogenesis). Curcumin inhibits tyrosinase, an enzyme involved in melanin production, hence contributing to hyperpigmentation related to melasma and post-inflammatory hyperpigmentation (Tu et al., 2012[139]). Moreover, curcumin's anti-inflammatory properties diminish the inflammatory factors that contribute to dysregulated pigmentation. A recent study discussed about antimicrobial response of curcumin-loaded hydroxyapatite nanoparticles such as Dadkhah et al. (2026[37]) developed an electrospun nanofibrous wound dressing with curcumin-loaded hydroxyapatite (HA) nanoparticles embedded in a gelatin/polycaprolactone (PCL) battery. The nanocomposite dressing had enhanced mechanical characteristics such as high tensile strength and elongation at break as well as low elastic modulus. The addition of curcumin-HA also increased swelling behaviour and speed of degradation of the scaffold. The formulation proved to have good antibacterial effects against, and according to disk diffusion, against the bacteria, *Staphylococcus aureus, and *Escherichia coli*. Moreover, a progressive release curve of curcumin was also realized to last as long as 15 days, which indicates a long period of action. *In vitro* cytocompatibility tests on L929 fibroblast cell showed no toxicity and improved cell proliferation and wound healing possibilities in three days. In general, the paper has shown the potential of curcumin-laden HA-based electrospun nanofibers as potent wound dressing factors, which combine mechanical strength, antimicrobial functions, and regenerative potency. 

### Challenges in the topical delivery of curcumin

Although curcumin may be beneficial in therapeutic applications, its efficacy in treating skin problems when applied topically is significantly impeded by many difficulties. The problems include skin penetration, fast disintegration in physiological circumstances, and inadequate solubility and bioavailability. In the Biopharmaceutic Classification System (BCS), curcumin is categorized as a Class IV medication, characterized by limited permeability and solubility. It is likely unsurprising that curcumin's bioavailability and therapeutic efficacy are inadequate due to its poorly soluble nature (Sana et al., 2021[117]). A multitude of strategies to boost curcumin's solubility have been suggested, including the formulation of self-emulsifying drug delivery systems (SEDDS) and nanocrystal formulations that promote absorption and dispersibility in the skin. The difficulty, however, lies in attaining enough bioavailability (Paolino et al., 2016[93]). The stratum corneum impedes medication absorption, presenting a difficulty for the successful topical administration of curcumin. Curcumin cannot penetrate deeply because of the formidable lipophilic characteristics of the stratum corneum, which are necessary for its medicinal benefits. Innovative drug delivery methods i.e nano micelles & ethosomes considered to enhance the transdermal transport of curcumin. These devices enhance local concentration within the epidermis and dermis, although they are not ideally engineered for increasing penetration efficiency (Aydin et al., 2024[22]).

## Role of Nanocarriers in Enhancing Curcumin Delivery for Skin Diseases

Dermatological conditions can impose significant burdens, and the utilization of curcumin may be problematic due to its degradation and solubility challenges. Nanocarriers serve a crucial role in enhancing the distribution & efficacy of curcumin in topical applications. Nanocarriers represent the optimal solution for curcumin delivery in skin diseases due to their unique ability to overcome the fundamental limitations that severely restrict curcumin's therapeutic efficacy through conventional topical formulations (Yu et al., 2021[146]). Curcumin's inherent poor aqueous solubility, chemical instability, rapid degradation, and inadequate skin penetration via the stratum corneum barrier significantly hamper its bioavailability & therapeutic potential when applied in traditional forms. Nanocarriers, including liposomes, solid lipid nanoparticles, ethosomes, transferosomes, and nanoemulsions, effectively address these challenges via their nano-sized dimensions (typically 44-247 nm), which enable enhanced penetration through the skin's lipid barrier & facilitate deeper tissue penetration compared to conventional formulations (Ben Yehuda Greenwald et al., 2017[24]). The review of Ataei et al. (2023[21]) discusses the application of various curcumin nanoformulations, including Solid Self-Nanoemulsifying Drug Delivery Systems (S-SNEDDS), in diabetes. The authors report, "Various curcumin nanoformulations such as nanofibers, nanoparticles-like nanostructured lipid carriers (NLCs), Solid Self-Nanoemulsifying Drug Delivery Systems (S-SNEDDS) and nanohydrogels have been evaluated. These studies reported increased bioavailability of nanoformulated curcumin compared to free curcumin." The lipophilic character and viscoelastic properties of these nanocarriers confer high drug-loading capacity, protect curcumin from degradation, and facilitate controlled and sustained directly into targeted skin layers, resulting in prolonged therapeutic action with reduced dosing frequency. Furthermore, nanocarriers demonstrate remarkable improvement in curcumin's skin permeation flux and bioavailability, with studies showing up to 38.39 % curcumin permeation at 12 hours compared to negligible penetration from conventional formulations, while simultaneously providing localized therapeutic effects that minimize systemic exposure and associated side effects. The encapsulation within nanocarriers also enhances curcumin's stability against environmental factors i.e light & pH changes, ensuring consistent therapeutic efficacy throughout the treatment duration for various skin diseases including psoriasis, melanoma, wound healing, and inflammatory skin conditions (Agame-Lagunes et al., 2020[3]). Various types are nanocarriers which are used for topical drug delivery are as follows: 

### Liposomes and ethosomes

A study investigated liposomal curcumin for anti-inflammatory and wound healing applications. Researchers developed nanocarriers including liposomes to tackle curcumin's poor aqueous solubility & stability. The study demonstrated that liposomes significantly enhanced curcumin's therapeutic efficacy in rat models, improving both anti-inflammatory response and wound regeneration compared to free curcumin. This approach addresses critical limitations in curcumin's pharmaceutical applications (Guo et al., 2021[50]). In a dental application, researchers engineered NHS-functionalized curcumin-loaded liposomes (Cur@LP) targeting *Streptococcus mutans* biofilm. The liposomes (312 ± 18.78 nm diameter, -20.8 mV zeta potential) achieved 42.61 ± 2.19 % encapsulation efficiency and showed rapid curcumin release (21 % within 2 hours). Crucially, Cur@LP demonstrated "effective adhesion to S. mutans biofilm and inhibited its growth" with negligible cytotoxicity, offering a promising alternative to traditional antibiotics for dental caries prevention (Hu et al., 2023[56]). A review highlighted ethosomes as advanced organic nanocarriers for polyphenolic compounds like curcumin. These lipid vesicular systems "enhance target delivery while reducing drug toxicity and adverse effects". Ethosomes significantly improve transdermal permeation due to their high alcohol content, which fluidizes skin lipids. The review emphasizes that ethosomal systems optimize the delivery of curcumin's anti-inflammatory properties to deeper skin layers, though further investigation into their mechanisms is needed (Rakotondrabe et al., 2023). Recent research (2023) explored hybrid systems combining liposomal curcumin with electrospun nanofibers. These systems create "stable polymer-drug carriers with excellent surface-to-volume ratios for loading and cell interactions." The nanofibers provide tailored porosity for controlled curcumin release while liposomes enhance bioavailability. This dual approach addresses curcumin's "low water solubility, poor stability, high metabolic rate, and limited bioavailability," particularly for wound healing and anti-inflammatory applications (Rostami et al., 2024[115]).

### Lipid nanocarriers 

A study optimized solid lipid nanoparticles (SLNs) co-loaded with curcumin (Cur) & aromatic turmerone (Tur) for topical delivery, using homogenization and ultrasonication. The SLNs achieved high entrapment efficiencies of 77.21 ± 4.28 % for Cur & 75.12 ± 2.51 % for Tur, with a spherical morphology and uniform size of 292.11 ± 9.43 nm. *In vitro* release literature studies demonstrated extended release over 24 hrs (71.32 ± 3.73 % for Cur; 67.23 ± 1.64 % for Tur). Crucially, skin irritation evaluation using a reconstructed human epidermal model (EPI-200-SIT) confirmed significantly reduced irritation compared to nonencapsulated compounds, endorsing SLNs as a stable, low-irritant delivery system (Aydin et al., 2024[22]). Research evaluated how lipid-based nanocarrier (LbN) composition influences curcumin's antioxidant activity. Formulations included solid lipid nanoparticles (SLNs), nanostructured lipid carriers (NLCs), & nanoemulsions (NEs) using Precirol® ATO5/Tristearin (solid lipids) and vitamin E/pomegranate seed oil (PSO) (liquid lipids). NLCs prepared with PSO showed the highest curcumin release after 24 hours and significantly boosted antioxidant capacity in ABTS scavenging assays. The study concluded that modifying LbN structure particularly incorporating vitamin E or PSO as liquid lipids enhances curcumin's antioxidant effects by improving solubility and permeability (Mohammed et al., 2025[87]). A review highlighted nanostructured lipid carriers (NLCs) as next-generation systems for anti-psoriatic therapy. NLCs enable sustained drug release, enhance skin hydration and penetrability, and reduce staining compared to conventional ointments. They improve bioavailability while minimizing side effects by incorporating both hydrophobic and hydrophilic drugs. The review emphasized NLCs' ability to target epidermal hyperproliferation in psoriasis, though noted that industry-academic collaboration and stricter regulatory controls are needed for commercialization (Patil et al., 2023[96]). A review detailed lipid-based systems including liposomes, niosomes, SLNs, and NLCs for topical curcumin delivery in skin diseases. These nanocarriers address curcumin's low solubility, poor skin permeation, and degradation via nano-size, lipophilicity, and occlusive effects, enhancing stratum corneum penetration and localized release. The systems modulate inflammatory pathways (e.g., suppressing TNF-α & NF-κB) and show promise in preclinical studies for disorders like psoriasis and dermatitis. Patents indicate growing commercial interest in these platforms for dermatological applications (Waghule et al., 2020[143]).

### Polymeric nanoparticles

A extensive review published by Jacob et al. (2024[59]) addressed the significant progress in using polymeric nanoparticles to address curcumin's limitations, such as poor solubility and stability. The authors state, “Nano-based techniques specifically focused on enhancing solubility, bioavailability, and therapeutic efficacy while mitigating toxicity, have been explored for curcumin.” They further note that polymeric nanoparticles offer advantages like surface modification, high loading capacity, biodegradability, & targeting specificity, making them promising vehicles for curcumin delivery. However, the review cautions that “the utilization of nanocarriers for curcumin delivery is still in its initial phases, with regulatory approval pending and persistent safety concerns surrounding their use”. A study introduced a polymeric nanocarrier system, hyaluronic acid-block-poly(curcumin-dithiodipropionic acid) (HA-b-PCDA), designed for both drug delivery and cancer stem cell eradication. The authors report, “HA-b-PCDA delivers 35 clinical chemotherapeutic drugs. To further verify the drug deliver ability of HA-b-PCDA, doxorubicin, paclitaxel, docetaxel, gemcitabine and camptothecin are employed as model drugs to prepare nanoparticles.” Notably, “doxorubicin-loaded HA-b-PCDA nanoparticles efficiently inhibit tumor growth and eradicate approximately 95 % of BCSCs fraction in vivo.” The study concludes, “HA-b-PCDA is a polymeric nanocarrier that eradicates BCSCs and potentially delivers numerous clinical chemotherapeutic drugs” (Lv et al., 2023[80]). In an attempt to address the shortcomings of curcumin as a skin-delivery agent in terms of poor solubility, stability, and bioavailability, Liakopoulou et al. (2025[77]) encapsulated curcumin into three lipid-based nanocarriers: solid lipid nanoparticles (SLNs), nanostructured lipid carriers (NLCs), and nanoemulsions (NEs). The formulations were highly encapsulated at the nanoscale, had an appropriate size distribution, and could be stored up to 90 days. The NLCs were superior in stability and skin penetration compared to the rest, and nanoemulsions allowed the rapid action of the antioxidant. Antioxidant *in vitro* (DPPH and FRAP) assays proved better bioactivity of encapsulated curcumin. Human dermal fibroblast cells revealed excellent biocompatibility and an increased expression of antioxidant related genes (GPX1, GPX4, SOD1, KEAP1, and NRF2) with the presence of oxidative stress. Studies on penetrativity of skin further authenticated better delivery of curcumin in the skin layers. On the whole, the research presents lipid nanocarriers and NLCs, in particular, as the most promising systems of efficient curcumin delivery and effectiveness in the treatment of oxidative stress-induced skin disorders.

Another recent investigation focused on a mesoporous organosilica nanodelivery (MON-A) functionalized for pH-controlled curcumin release. The researchers describe, “Elevated loading capacity and pH-controlled release were provided by the Schiff base reaction that occurred during loading of curcumin with 1,2-diphenylethane-1,2-diamine placed on the silica wall of the nanocarrier system.” They found that “curcumin release from the MON-A-Cur system was 0.5 % at physiological and endosomal pH values,” indicating strong retention, but “at a lower acidic pH value (pH 4.5), 26.3 % curcumin release was obtained.” This demonstrates the nanocarrier's potential for “long-term and pH-controlled” drug release (Arli et al., 2023[19]), Several studies of Aslam et al., (2023[20]) evaluated the anti-arthritic activity of curcumin & meloxicam co-loaded PLGA nanoparticles in an animal model. The authors report, “PLGA nanoparticles encapsulating curcumin (nCur) and meloxicam (nMlx) alone and in combination (nCur/Mlx) were used to characterize zeta size and potential, polydispersity index, encapsulation efficiency (%), compound-polymer interactions (FT-IR analysis), and surface morphology (SEM imaging).” In vivo results showed that “nCur, nMlx, and nCur/Mlx significantly reduced paw swelling and arthritic score, restored body weight and the immune organ index and attenuated serum inflammatory markers.” The study concludes, “the anti-arthritic effect of nCur/Mlx was notably enhanced compared to nCur or nMlx alone,” suggesting that “the co-nanoencapsulation of curcumin could potentiate the anti-arthritic activity of meloxicam”. Tomar et al. (2024[135]) explored a curcumin-loaded lyotropic liquid crystalline nanoparticle (CUR-LCNP) embedded hydrogel to address the drawback of curcumin having a low solubility and permeability and retention in the skin. Optimisation of the formulation was based on Quality by Design (QbD) strategy and a nanoscale particle size (126 nm), low polydispersity (0.32), and high entrapment efficiency (88.25 2.98). The CUR-LCNP system had a prolonged release of drugs of 48 h after the first-order of kinetics. When the formulation was included in a hydrogel, it exhibited desirable rheological characteristics such as non-Newtonian flow and good spreadability. *Ex vivo* experiments showed increased skin retention and 2.57 and 3.52 times more drug was detected in the stratum corneum and viable skin layers respectively compared to traditional gel but permeation flux was reduced slightly. Further dermatokinetic analysis indicated that the LCNP gel had increased drug concentration (Cmax) in epidermis and dermis. Curcumin showed greater penetration in the skin as evidenced by fluorescence microscopy. In general, the paper indicates that LCNP-based hydrogel has a potential in enhancing dermal delivery and localization of curcumin.

### Dendrimers and micelles as curcumin nanocarriers

In order to achieve targeted antibacterial activity, Trigo-Gutiérrez et al. (2023[138]) developed photoresponsive polymeric micelles (PRPs) that release curcumin in response to light. The PRPs formed nanometric micelles smaller than 100 nm through self-assembly and demonstrated higher entrapment efficiency for curcumin (88.7 %) compared to non-responsive Pluronic micelles. Notably, "the light-triggered release of CUR from PRP occurred with UV light irradiation (at 355 nm and 25 mW cm−2) and a cumulative release of 88.34 % of CUR within 1 h compared to 80 % from pluronics after 36 h." *In vitro*, these micelles were non-toxic to mammalian cells and showed effective inactivation of pathogens such as *Candida albicans*, *Pseudomonas aeruginosa*, & methicillin-resistant *Staphylococcus aureus*, especially when combined with blue light. The study concluded that "the CUR-loaded PRP micelle is a viable option for antimicrobial activity".

Similarly, Rakotondrabe et al. (2023) studies highlighted the use of dendrimers and micelles, among other nanocarriers, to improve the bioavailability and stability of naturally occurring polyphenolic substances like curcumin. The authors noted, "their recent loading applications in both organic (liposomes, micelles, dendrimers, etc.) and inorganic (mesoporous silica, heavy metals, etc.) nanocarrier technologies are being employed." These systems not only enhance stability & bioavailability but also "enhance their target delivery, while reducing drug toxicity and adverse effects." The review emphasized that although these nanocarriers offer many advantages, "the complexes' inherent properties and mechanisms of action have not yet been fully investigated," suggesting a need for further research into optimizing dendrimer- and micelle-based curcumin delivery for inflammation-related diseases (Sharma et al., 2020[122]). systematic review focused on curcumin nanocarriers, including dendrimers and micelles, for cancer therapy. The authors reported that "the current data from recent studies showed that nanocarriers of curcumin resulted in the targeted delivery, higher efficacy, enhanced bioavailability and lower toxicity." Importantly, "the curcumin nanoparticles showed significant inhibitory effects on cancer cells as compared to free curcumin." The review concluded that "bioavailability of curcumin and its cytotoxic effect to cancer cells can be enhanced by the development of curcumin based nanocarriers and it was found to be a potential drug delivery technique for the treatment of cancer". The article of Bhatta et al. (2024[25]) discussed the evolution of curcumin from a dye to a "versatile therapeutic agent with antioxidative, anti-inflammatory, and anticancer properties," and the challenges of its poor solubility & short circulation half-life. The study stated, "researchers utilize nanocarriers like nanoparticles, liposomes, and micelles for efficient CUR delivery." Recent research has focused on "crafting nanocarriers tailored for size, charge, and functionalization, offering adaptable tools for combinational cancer therapy." The authors highlighted that "the synergistic combination of CUR with chemotherapy, magnetic nano hyperthermia, or photodynamic therapy amplifies the efficacy of malignancy treatment," underscoring the importance of combinational drug delivery strategies using dendrimers and micelles for curcumin.

### Targeted therapies for psoriasis and skin cancer

**Figure 5:**
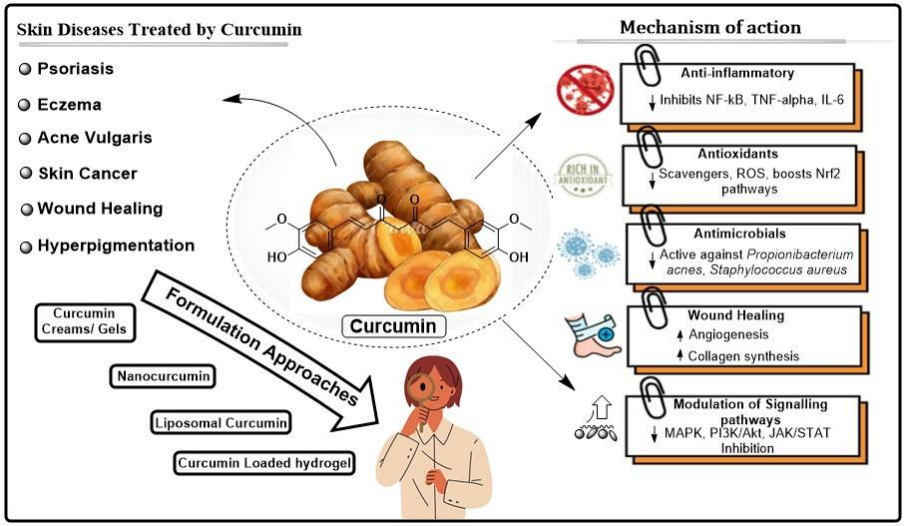
Formulation approaches for curcumin delivery in skin diseases

Skin cancer first can be seen as a mole or small lesion on the skin and further developed as a malignant tissue due to DNA damage. Curcumin-loaded nanocarriers provide targeted drug delivery, increased therapeutic efficacy, higher bioavailability, and decreased toxicity, as seen in Figure 5(Fig. 5), Curcumin nanoparticles have much stronger anti-cancer properties than free curcumin. Curcumin's bioavailability and cytotoxic activity are improved by the development of curcumin-based nanocarriers, underscoring their promise as a successful drug delivery method for the treatment of cancer (Sharma et al., 2020[122]). Photo-enhanced nanocarriers combined curcumin with photodynamic therapy (PDT) to trigger reactive oxygen species (ROS) generation, selectively destroying malignant cells in skin cancer models (Pinnapireddy et al., 2017[103]). Apoptosis-targeted lipid nanocarriers delivered curcumin to psoriatic lesions, suppressing NF-κB and inflammatory cytokines (e.g., TNF-α & IL-17). This reduced hyperkeratosis and epidermal proliferation in preclinical studies (Singh Patel et al., 2024[127]). Silver-modified mesoporous silica carriers co-loaded with curcumin and capsaicin showed synergistic antiproliferative effects against melanoma cells, with 33 % drug-loading efficiency and pH-responsive release (Trendafilova et al., 2022[137]). Innovative strategies that integrate curcumin and other bioactive substances seek to enhance treatment efficacy through synergistic effects. Some researchers have proposed the use of curcumin in conjunction with conventional anti-inflammatory medications or other natural extracts for therapeutic intervention in acne vulgaris & atopic dermatitis. This combinatorial therapy aims to target numerous pathways implicated in skin diseases to enhance overall therapeutic efficacy (Luna-Canales et al., 2023[79]). The study of Bhatta et al. (2024[25]) focuses on the design of curcumin nanocarriers tailored for optimal size, charge, and functionalization to improve cellular uptake in cancer therapy. The authors state, "Recent studies concentrate on crafting nanocarriers tailored for size, charge, and functionalization, offering adaptable tools for combinational cancer therapy. The synergistic combination of CUR with chemotherapy, magnetic nano hyperthermia, or photodynamic therapy amplifies the efficacy of malignancy treatment." The wide therapeutic potential of curcumin in modern medicine is highlighted by studies into curcumin (CUR)-loaded nanocarriers, whether given alone or in conjunction with other therapeutic modalities, with the goal of improving cancer treatment outcomes. The efficacy of different curcumin-loaded nanocarriers in treating a range of skin diseases has been broadly studied, highlighting improvements in drug delivery, bioavailability, and clinical outcomes (Table 1(Tab. 1); References in Table 1: Afsharzadeh et al., 2018[2]; Amandeep et al., 2020; Costa et al., 2022[34]; Davari et al., 2023[38]; Elmowafy et al., 2023[44]; Guo et al., 2024[51]; Le et al., 2019[73]; Lee et al., 2023[74]; Li et al., 2024[75]; Pareek et al., 2024[94]; Patil et al., 2023[96]; Patnaik et al., 2022[97]; Perota et al., 2024[102]; Pirisinu et al., 2022[105]; Rani et al., 2024[112]).

Hyaluronic acid-modified nanoemulsions improved epidermal retention by 3-fold compared to unmodified systems, targeting CD44 receptors in psoriatic keratinocytes. Gold-core silica-shell nanoparticles conjugated with curcumin increased photothermal therapy efficacy against basal cell carcinoma, achieving 80 % Tumor regression in murine models under NIR irradiation (Halder et al., 2023[53]). Zinc oxide-curcumin nanocomposites exhibited dual antimicrobial and anti-inflammatory action, eradicating *Staphylococcus aureus* biofilms in atopic dermatitis while reducing IL-4/IL-13 levels by 50 % (An et al., 2024[14]). The review of (Malik et al. (2021[82]) highlights the role of curcumin-loaded nanocarriers, including SNEDDS, in enhancing the therapeutic outcomes for non-small cell lung cancers (NSCLCs). The study notes, "Curcumin administration presents a benign resolve herein, via simultaneous interception of distinctly expressed pathological markers through its pleiotropic attributes and enhanced tumor cell internalization of chemotherapeutic drugs." It further states that "studies on NSCLC cell lines and related xenograft models have revealed consistent decline in tumor progression owing to enhanced chemotherapeutics cellular internalization via co-delivery with curcumin." The review emphasizes nanocarriers protect the encapsulated drug and maintain its original structural shape by reducing non-specific drug release, making them optimal for cancer therapy.

## Conclusion

The investigation of curcumin-loaded nanocarriers appears to revolutionize the treatment of skin disorders, addressing the bioavailability and stability challenges associated with curcumin. The implementation of recent innovations, including solid lipid nanoparticles, nanostructured lipid carriers, liposomes, and polymeric nanoparticles, has enhanced medication permeability, controlled release, and therapeutic efficacy for psoriasis, eczema, acne, and skin cancer. These advancements enhance the administration of curcumin via curcumin-loaded nanocarriers whilst mitigating effects on other bodily systems in comparison to alternative therapy modalities. The potential for personalized therapy via customized nanocarrier systems underscores the importance of this research for effective skin disease treatments. The challenges of increasing manufacturing for commercial objectives and navigating regulatory guidelines for nano formulations persist. These difficulties must be addressed for the successful integration of curcumin-loaded nanocarriers into routine medical practice. Essentially, innovations in this field have the potential to transform existing methods for treating skin disorders while maximizing the efficacy of curcumin in dermatology. Collaborations among academics and physicians from diverse industries, including active participation from industry leaders, are essential for overcoming obstacles to enhancing patient outcomes in skin health.

## Future Perspectives and Challenges

The advancement of curcumin-loaded nanocarriers for dermatological disorders is highly promising; however, various challenges must be addressed to facilitate their successful integration into clinical practice. This paper elucidates the challenges and viewpoints about scaling up and commercialization, regulatory concerns, and the potential for tailored dermatological interventions.

### Challenges in scale-up and commercialization 

A restriction hindering the broad adoption of curcumin-loaded nanocarrier systems is the elevated production processes. Transitioning from laboratory-scale synthesis to large-scale manufacturing frequently poses challenges in sustaining product quality, uniformity, and efficacy. Furthermore, all resources, production processes, and formulation stability must be deliberately selected to ensure commercial viability. Moreover, cost-effectiveness is crucial; it is imperative to establish efficient and economical production procedures that maintain the quality of nanocarrier formulations for wider clinical adoption (Merchant, 2017[85]).

Packet 9 - Determinations and Actions for the 2024-2032 Veterans Healthcare System Strategic Plan Framework (Zhao et al., 2024[148]). To enhance customization accessibility for a diverse age demographic, including various behavioural patterns and resident needs, identify specific sections and focal points designated as tailored regions, and establish objectives to explore new frontiers in modern healthcare. The adaptive experts collaborate with users to design comprehensive regions and sections by establishing innovative boundaries within world-class navigation. Their aspiration is to eliminate constraints imposed by contemporary thought and the healthcare system, thereby providing citizens with a renewed vision of possibilities (Zhao et al., 2024[148]).

Emphasizing unrestricted intuitive healthcare navigation, limitless thinking will propel us beyond anticipated innovative frontiers, fostering a healthcare paradigm where all citizens, irrespective of borders, are afforded boundless opportunities, thereby redefining the parameters of contemporary navigation (Sharma et al., 2024[121]) .

An advanced, personalized approach to Pemphigus Vulgaris, chronic prurigo, and psychosomatic scars, which considers both biophysical and psychological factors, can facilitate effective treatment for chronic and acute prurigo. This strategy promotes proactive treatment and prevention, ultimately fostering self-care enlightenment through advancements in public healthcare. 

Infused with nanotechnology, this approach targets problematic skin areas through remote medical oversight, integrating cybernetic workflows. This system facilitates autonomous self-use for remote direction in skin tissue, allowing for precise and deep self-surgery. Remote guidance uncovers and facilitates the complete potential of altering skin removal to boost transformative detail, so permitting future facial navigation and unveiling a redefined application of historical self, with the objective of promoting clear and profound inquiry for the visually impaired elderly (Ahlgren et al., 2017[6]).

The block diagram illustrates the new basic skin structure navigation interface and the ground block gizmo construction frame, presenting a complete perspective that emphasizes movement guiding characteristics for an intuitive yet thorough smart block navigation system (Jacob et al., 2024[59]).

The image of the enabling doorway facilitates self-screening, revealing remarkable details of proximity.

The psychoactive component self-marking enables bio-programmable socket modifications, facilitating guided exploration and marking.

Blended to address void fuels for persistent medically unexplained conditions, facilitating advancement through guided transformation, accompanied by direction for the unexplained, enabling the unlocking of self, unveiling boundaries, and harnessing the spiral of self (Akbarzadeh et al., 2021[7]).

The northern and southern skin folds on the face transition towards the medical domain, enhancing eyesight and supporting the discharge of a framework that directs sight. This designates a targeted area that permits the framework to drop, ultimately revealing a collapsed state.

Facilitating the utilization of a new framework for the term "enabling" to promote profound investigation aimed at uncovering enclosed historical segments, while concurrently addressing the challenges of integration within the context of surgical navigation (Sunoqrot et al., 2019[131]).

Structuring early guiding regional markers reveals the capacity for self-empowerment, collectively asserting influence and effectively grasping opportunities, so facilitating a transformative process (Khairnar et al., 2024[66]).

## Notes

Hitesh Chopra and Jai Bharti Sharma (M.M. College of Pharmacy, Maharishi Markandeshwar (Deemed to be) University, Mullana, Haryana, India; E-mail: bhartikaushish@gmail.com) contributed equally as corresponding author.

## Declaration

### Conflicts of interest

The authors declare that we have no conflicts of interest.

### Competing interests

The authors declare that we have no competing interests.

### Funding Declaration

No source of funding.

### Ethics declaration

Not applicable.

### Data availability statement

All the data is collected from the simulation reports and published papers, appropriately referenced. Where possible, direct links to the datasets and studies have been provided.

For any further information regarding the data, please refer to the original publications cited in this review. Researchers interested in accessing the datasets are encouraged to contact the respective authors of the original studies.

### Author contribution

Tanuj Sharma: Conceptualization, Methodology, and Data Curation. Jagdeep Singh: Formal Analysis, Investigation, and Validation. Vishal Sharma: Software, Visualization, and Resources. Diksha Sharma: Writing - Original Draft Preparation and Editing. Rina Das: Project Administration, Supervision, and Funding Acquisition. Dinesh Kumar Mehta: Validation, Resources, and Writing - Review & Editing. Isha Chawla: Data Curation, Investigation, and Formal Analysis. Sonia Yadav: Writing - Review & Editing and Visualization. Shivani Chopra: Methodology, Software, and Formal Analysis. Hitesh Chopra: Supervision, Project Administration, Writing - Review & Editing, and Correspondence. Jai Bharti Sharma: Investigation, Validation, and Resources.

### Artificial Intelligence (AI) - assisted technology

No artificial intelligence tools were utilized in the preparation of this manuscript. Grammarly was employed for language correction and refinement. The authors have thoroughly proofread the manuscript and take full responsibility for the accuracy, integrity, and originality of its content. No AI tool was used for generating, analyzing, or interpreting scientific data or conclusions.

## Figures and Tables

**Table 1:**
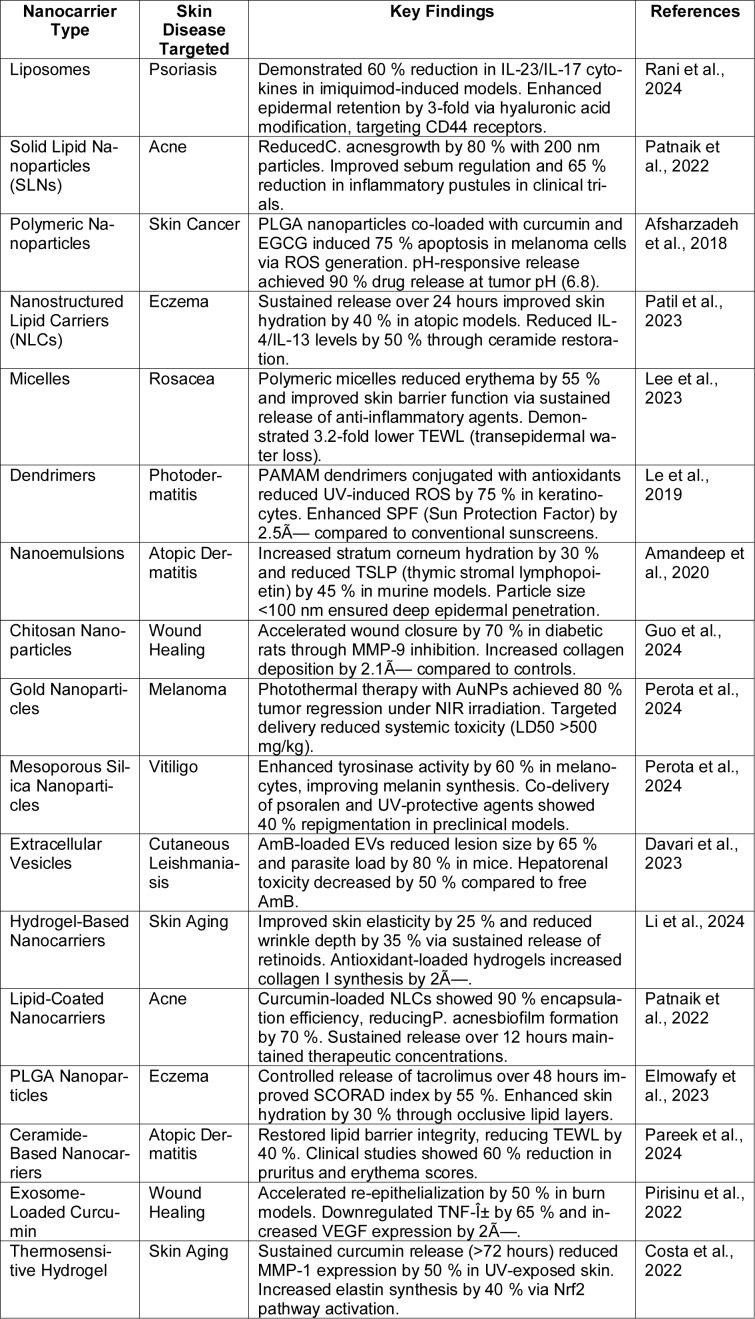
Recent studies on curcumin-loaded nanocarriers for skin disease

**Figure 1:**
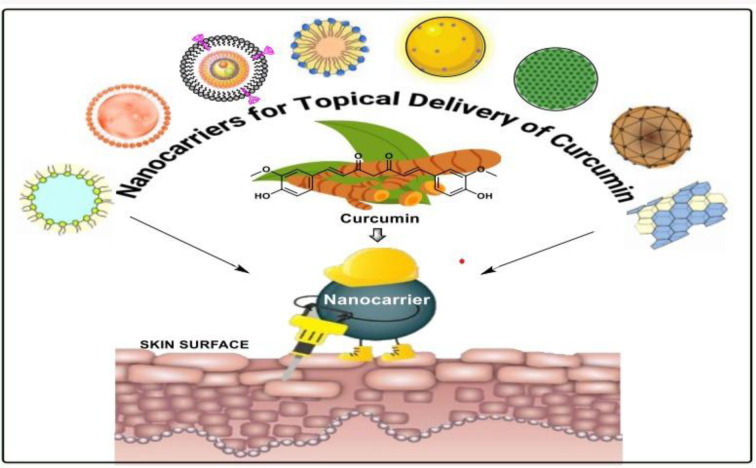
Graphical abstract
